# Stigma in Mothers of Deaf Children

**Published:** 2015-03

**Authors:** Hossein Ebrahimi, Eissa Mohammadi, Mohammad Ali Mohammadi, Akbar Pirzadeh, Hamzeh Mahmoudi, Ismail Ansari

**Affiliations:** 1 *Department of Psychiatric Nursing, School of Nursing and Midwifery, Tabriz University of Medical Sciences, Tabriz, Iran. *; 2 *Department of Nursing, School of Medical Sciences, Tarbiat Modarres University, Tehran, Iran.*; 3 *Research Committee member and PhD Candidate in Nursing, School of Nursing and Midwifery, Tabriz University of Medical Sciences, Tabriz, Iran.*; 4 *Department of Otorhinolaryngology, Fatemi Hospital, Ardabil University of Medical Sciences, Ardabil , Iran. *; 5 *Ardabil education and training organization, Ardabil, Iran.*; 6 *Audiology Clinic, Welfare Organization, Ardabil, Iran.*

**Keywords:** Children, Hearing loss, Mother, Stigma

## Abstract

**Introduction:**

A deaf child creates a feeling of stigma in many hearing parents. Stigma in mothers can have a negative impact on a child’s treatment and rehabilitation process. Therefore, this study was conducted to evaluate the extent of stigma in mothers with deaf children.

**Materials and Methods:**

This descriptive, cross-sectional study was conducted in 2013 among 90 mothers with deaf children. The data-collection instrument included the stigma scale in the mothers of children with disabilities. The reliability and validity of the instrument were confirmed through content validity and Cronbach’s alpha coefficient (α=86%), respectively. Data were analyzed using SPSS-15 software.

**Results:**

Results showed that most mothers suffer from stigma due to having a deaf child. The mean stigma score was 96.48 ±27.72. In total, 24.4% of mothers reported that they had received strange and mocking looks; 72.2% regarded child deafness as a sign of divine retribution; and 33.3% felt ashamed of their child’s deafness. There was an inverse relationship between the mother’s level of education and mean stigma scores (P<0.033). The stigma score was higher in mothers who were living independently of their relatives (P<0.029). The mean stigma score in mothers of children with a cochlear implant was lower than that of mothers of children with earphones (86.70 vs. 99.64), and this difference tended towards significance (P=0.057).

**Conclusion:**

This study showed that half of all mothers with deaf children were scorned and felt ashamed of having a deaf child in the family because of the stigma. The majority of mothers with deaf children felt stigmatized, and only their education and residency status affected this issue. The mothers of cochlear-implanted children perceived less stigma. Due to the various social and psychological problems caused by hearing impairment, it is necessary to consider the emotional health and psychological state of the mothers in addition to rehabilitation programs and standard services for the children themselves.

## Introduction

Deafness is one of the most significant disabilities at birth (1,2). Approximately 1–3 children per 1,000 are born with moderate-to-severe hearing loss, accounting for 2–4 newborns per 1,000 hospitalized in an intensive care unit (ICU). The prevalence of deafness is higher than that of other birth disorders screened for at the time of birth (3-7). In Iran, the prevalence of deafness is reported to be 4.7 per 1,000 live births in provincial capitals (8). 

Child deafness is not only a medical problem, but also a social problem (9,10). Several studies have shown that although deafness is not a disability, individuals affected are often labeled as disabled (11). Historically, society has attributed stereotyped behaviors to deaf people, and has treated these individuals with prejudice; notably in the works of Aristotle and other prominent Greek philosophers. Even today, hearing impairment and its associated physical disability is regarded as a defect in social interaction, and hearing loss is considered a stigma (12,13). Today, hearing-loss-induced stigma is common in most developed societies (14). 

Hearing-impaired children and their families form a unique group different to that of healthy children (9,11). Evidence suggests that the physical and psychological needs of disabled children and the time and energy required to take care of them are unique challenges for mothers (15). Depending on their personality, mothers feel responsible and sometimes guilty, often leading to a need to obtain more information on child disability and extreme support solutions (16). Evidence from around the world suggests that deafness in children can exacerbate their negative self-attitudes and may even lead to the social isolation of their parents. Although mothers are able to cope with their child’s hearing loss, most are upset with the attitudes of individuals and the society at large towards their child’s hearing loss (17). Some mothers with deaf and disabled children become socially isolated and limit their social relationships, and may be more vulnerable to post-traumatic stress disorder (16)(15). Furthermore, some mothers may even perceive their child’s use of a hearing aid as a reason for stigma (10). 

Studies have shown that deaf individuals are not always treated well, and that their abilities are not generally recognized in society; indeed, sometimes deaf people are thought to have a low IQ because of their hearing problems (11,18). In addition, people’s judgment of this group is always associated with negative prejudices (9). Negative consequences including feelings of shame, depression, low self-esteem, and social isolation may be directly related to the internalization of relatives’ stigma (19). In a study conducted by Green to investigate the effects of perceived stigma on social and emotional outcomes of mothers and children, it was revealed that the mother’s perceptions and internalization of stigmatizing beliefs about children, as well as the objective burden of caring for such a child, increased her stress. Further, the children of mothers with higher feelings of stigma have less interaction with their peers in an informal environment at home and with neighbors. 

Perceived stigma in mothers with disabled children affects both subjective and objective aspects of stress (20). In particular, the stigma of a deaf child limits the families in accepting the problem, searching for treatment, obtaining social and supportive services, and in the rehabilitation process (14,21,22), and is the most important factor in deafness denial and audiometric assessment rejection (13). 

It is clear that stigma is a real socio-psychological phenomenon which involves different groups with special needs, while its associated costs may be irrecoverable (22). Undoubtedly, a deeper understanding of stigmatization is essential in mothers with deaf children so that they may successfully cope with a deaf child and develop care plans and start effective rehabilitation processes. According to a literature review of the Springer, Science Direct, Elsevier, and Pubmed databases using the keywords, ‘deaf child’, ‘mother’, and ‘stigma’, no studies have been published in the nursing or medical literature assessing the stigma of mothers with a deaf child. Thus, this study was conducted in order to investigate stigma in mothers of deaf children. 

## Materials and Methods

This cross-sectional study was carried out in 2013. Through a census sampling method, mothers (N=90) with a congenital deaf child, diagnosed for at least 6 months, were included in the study. Mothers who had a child with multiple disabilities and mothers with more than one disabled child were excluded. A two-part questionnaire was used as the evaluation instrument. The first part included the demographic characteristics of the mothers and children, including age, education, occupation, positive family history of deafness in mother, child’s gender and age, as well as the mother’s perceived level of shame in having a deaf child, resentment of other children, sadness caused by the pity of others, and the child’s inability to perform regular work. The second part of the questionnaire evaluated the perceived stigma for mothers of children with disabilities using the stigma scale. This is a self-reporting scale designed by Dehnavi et al. using the Link and Ferran Theory and interviews with parents and specialists (19). This instrument consists of 48 questions which are arranged on a Likert scale (from 1 for ‘never’ to 5 for ‘always’) and assesses two perceptional and behavioral aspects. These aspects include:

1) mother’s belief in others’ stereotype of a deaf child (e.g. “others think that my child is always in need of protection” or “others think that having this child is a sign of divine punishment”);

2) mother’s belief in her stereotype (e.g.“I think that my child is always in need of protection” or “I think that having this child is a sign of divine punishment”); 3) behaviors based on social isolation and discrimination (e.g. “People stare at us with a strange look” or “having such a child is an embarrassment.”). These scores ranged from 48 to 240. Initially, a score of 1, 2, 3, 4, or 5 was considered for each question, respectively, and then the ratio of the total score was calculated for each sample and the stigma score for each mother was determined using the formula: 

[Stigma score=(the obtained score- minimum obtainable score)÷score range ×100].

A score of 48 was taken as the threshold for a the lack of stigma (19), and was thus chosen as the criterion for comparison of mean stigma scores. 

The reliability and validity of the instrument were previously reported to be desirable in the study of Dehnavi et al. (19). In the present study, the content validity of the instrument was reviewed by a team consisting of five faculty members, and the content validity index (CVI) (Waltz and Bausell) was used to assess the numerical value of the content validity (23). The average evaluation obtained was 3.5. After applying corrective views, Cronbach’s alpha coefficient was used to determine reliability (α=86%). Before distribution of the questionnaires, the project generalities, objectives of the study, methodology, details of the individuals and organizations benefiting from the study, as well as the anonymity of individuals and confidentiality obligations were explained to all mothers. After signing an informed consent, mothers were given the questionnaire for completion, with the help of a language specialist if necessary. The completeness of each questionnaire was checked before data entry. Statistical analysis was performed using SPSS-15. The Kolmogorov-Smirnov test was used to review normal data. The mean and standard deviation were reported for quantitative variables such as mother’s age, child’s age and stigma scores. A T-test and analysis of variance (ANOVA) were used to examine the differences between mean stigma score based on the demographic characteristics of the mother and child. A one-sample t-test was also used to examine the difference between the mean stigma score with the standard stigma score of 48. P-values less than 0.05 were considered statistically significant.

## Results

The mean age of the mothers was 29.97±5.91 years, with a range of 18–39 years. In total 15.6% of participants were aged more than 35 years during pregnancy, 45.6% lived in their own private house, and 30.3% had a positive family history of deafness. The mean age of the deaf children was 4.10±1.44 years. Most mothers (83.1%) were reluctant to allow their child to marry another deaf person. Other results were as follows; 52.2% of mothers did not want to have another child because of their deaf children; 24.4% reported receiving strange looks and mocking; 72.2% regarded child deafness as a sign of divine retribution; and 77.8% claimed that people compared their child with other healthy children.

The mean stigma score with standard deviation was 96.48±27.72, with a range of 48.75–165.75. Results of the one-sample t-test performed to check whether there is a stigma among mothers with deaf children showed that the difference between the obtained mean (96.48) and the expected mean (48) in the population was 48.4833 (P<0.001).

The results also showed that the mean stigma score in mothers with cochlear-implanted (CI) children was less than that in mothers whose child used a hearing aid (HA) (86.70 vs. 99.64) and that this difference tended towards significance (P<0.057). The score reflecting the stereotypes of others due to a deaf child in the family was less in mothers of CI children compared with mothers of children who used hearing aids (37.09 vs. 45.45; P<0.014). Table 1 shows the mean stigma and its components in the mothers of children with cochlear implants and hearing aids. 

**Table 1:** Frequency and mean of stigma and its components in mothers of children with CI and HA

**Stigma and its components**	**Number (Percent)**	**Mean and SD**	P*
Mother belief in her own Stereotypes			0.291
CI HA	22 (24.4) 68 (75.6)	34.00±10.20 36.80±10.94	
Mother’s belief in others’ Stereotype			0. 014
CI HA	22 (24.4) 68 (75.6)	37.09±12.49 45.45±13.94	
Avoidance behaviors			0. 546
CI HA	22 (24.4) 68 (75.6)	16.81±8.03 17.86±6.72	
General stigma			0.0 57
CI HA	22 (24.4) 68 (75.6)	86 .70±27.28 99 0.6 4±27.31	

Results showed that 25.6% of mothers were ashamed of having a deaf child, and this varied based on the mother’s level of education. Mothers with less education were more ashamed (59.3 vs. 40.7%; P<0.025). The average stigma score in mothers with a sense of embarrassment of having a deaf child (120.70 vs. 88.16) was greater than others (P<0.001). In addition, 31.1% of mothers envied other children, 42.2% of mothers pitied their own child, while the majority of mothers (72.2%) believed that others thought their child needs pity. A total of 47.8% of mothers were upset about others’ pity for their child and their mean stigma score was higher than others (P<0.001); while 84.4% of mothers believed that child deafness was a tormenting problem (Table. 2).

**Table 2:** Mean and standard deviation of fundamental questions of stigma perceived by study sample

**Options **	**Number (Percent)**	**Mean and SD**	**P ** *
Feeling ashamed of having a deaf child			0.001
Yes No	23 (25.6) 67 (74.4%)	120.70±27.67 88.16±22.51	
begrudging others’ children			0.307
Yes No	28 (31.1) 62 (68.9)	100.5 ±22.74 94.65±29.69	
Troubled by others’ pity for their child			0.001
Yes No	43 (47.8) 47 (52.2)	108.19±26.61 85.77±24.40	
Child deafness can be a tormenting problem			0.018
Yes No	76 (84.4) 14 (15.6)	98.48±29.12 85.60±14.83	
Deaf children can work in normal jobs			0.017
Yes No	76 (84.4) 14 (15.6)	92.71±25.18 116.96±32.70	

* Independent T-test

The mean perceived stigma score in mothers with a positive family history of hearing loss was less than in others (91.6 vs. 98.76), but this difference was not statistically significant. The stigma score in mothers with a deaf son (97.09±27.54) was greater than that in mothers with a deaf daughter (95.81±28.23), but this difference was not significant (Table 3). 

**Table 3:** Mean and SD of stigma based on demographic characteristics

**P**	**Mean± SD**	**Number (%)**	
0.333*			Child age
	97.26±26.94 94.62±28.53 113.75±18.90	27(30 ) 58(64.4 ) 5(5.6 )	6 months -3year 3-6 year ˃6 year
0.776*			Mother age
	92.43±24.07 98.66±25.31 95.85±30.84 101.39±32.64	25( 27.8 ) 23(25.6 ) 28(31.1 ) 14(15.6 )	18-25year 25-30 year 30-35 year ˃35 year
0.033*			Educational status
	113.58±20.31 98.08±29.32 85.11±22.92 104.41±27.59 83.38±26.21	6 (6.7 ) 27 (30 ) 19 (21.1 ) 27 (30 ) 11 (12.2 )	Illiterate Elementary Secondary Diploma Higher education
0.029*			Residence Status
	103.62±7.14 95.91±28.93 84.87± 24.23	41 (45.6 ) 25(27.8 ) 24 (26.7 )	Private house Tenancies Living in relatives house
0.136**			Occupation
	103.91±8.99 95.95±28.25	6(6.7) 84(93.3)	Employee Housewife
0.248**			Setting
	101.2±32.14 95.03±26.32	21(23.3) 69(76.7)	Rural Urban
0.613**			Family history
	91.60±28.21 98.76±27.66	27(30.3) 62(69.7)	Yes No
0.483**			Child sex
	95.81±28.23 97.09±7.54	43(47.8) 47(52.2)	Female Male

* One-way ANOVA test

** Independent t-test

The average stigma score in illiterate mothers was higher than in other groups (113.58±20.31; P<0.033). 

Furthermore, the stigma of mothers living in nuclear families was greater than in other groups (103.62 ± 7.14; P<0.029). 

## Discussion

Stigma is a set of beliefs and behaviors that are activated by labeling and lead to social exclusion and isolation. Stigma not only affects the labeled individual but may also be passed on to their relatives and may have the same negative consequences in relatives (24).

The findings of this study showed that there is a stigma in mothers with deaf children, and indicates that this group of mothers is vulnerable to psychological issues. Unfortunately in Iran, there is lack of knowledge concerning children with hearing loss (25). A deaf child in the family is very susceptible to the negative attitude of the family, relatives, friends, and neighbors, and exposes the family to pessimism, doubt, and suspicion (11). In the study of Pelchat et al., many of the mothers also reported that there is no positive feedback for their child from society and that most people look at the condition as a stigma (26). Bat-Chava also showed that deaf people constitute a small group of society, and that judgment of this group by society is unfortunately associated with negative prejudices; thus child deafness often causes stigma and a sense of mourning in parents (27). In the study by Kumar and Lalita, the mean score of attitude of mothers with deaf children (297.6) was lower than that of mothers of healthy children (315.4), but this difference was not statistically significant (28). In the current study, a part of the stigma perceived by mothers may be due to the society’s ignorance of deafness or a reflection of the mother’s regret for the loss of a healthy baby.

Results of the current study indicate that stigma in mothers with CI children is less than that of mothers whose child uses a hearing aid. This finding is also consistent with the results of the study conducted by Span et al. who reported that socio-mental parameters of parents of CI children and parents of children with hearing aids are different (29). However, these findings are inconsistent with results of the study by Kuitner et al. and Burger et al. These studies showed that the parents of CI children experience greater mental distress than the parents of hearing children (30) and children who use hearing aids (31). Perhaps this is because significant progress may be occur in the child’s communication skills after CI, and this improvement is predominantly in the field of ​​social interaction, feelings of happiness, lack of isolation and communication like other family members; and that parents consequently feel greater reward for their efforts.

The stereotypical thinking others about deaf children was lower in mothers with implanted CI child. The image of a deaf child in society is linked to terms such as abnormal, backward, disabled etc. and these labels are sufficient to socially marginalize the child and his/her family (11). 

This study showed that mothers have different feelings towards a deaf child, and the majority of mothers regard the child's deafness as a sign of divine retribution. In Frank’s study in Nigeria, 72.97% of mothers felt that child deafness was a sign of God’s punishment for bad deeds and 78.38% felt pity for the deaf child (2). In the study by Gilbey, mothers also regarded child's deafness as a penalty for their sins (32).

In the current study, 25% of mothers felt shame or distress for their child’s deafness. These results are consistent with other studies. In the study of Pelchat *et al*. the majority of mothers felt ashamed of having a deaf child (26). Various studies also demonstrated that shame is a consequences of child deafness (33). While mothers are proud of having a healthy child, having a deaf child may cause feelings of shame and embarrassment, and lead to social exclusion and isolation. These consequences can be induced by the internalization of stigma in mothers (2, 20). It might be interpreted that when a mother’s expectation of having a healthy child is compromised, she experiences feelings of embarrassment and shame.

Most mothers believe that people feel pity for their child and look at their child with pity and compassion. The results of the study by Narimani et al. also showed that due to the deafness of the child, mothers face new challenges including the reaction of relatives (34). Jackson et al. also showed that other people usually have little understanding of the issue and that their response is mostly in the form of pity. Thus, mothers often experience some form of loneliness and social isolation, which reduces their level of social and intimate relationships (35). 

In this study, there was a significant decrease in the mean stigma score of mothers with a higher level of education; illiterate mothers or those with less education were more ashamed of their child’s deafness. These findings are in contrast with the results of Daramadi who showed that parents with an academic education experienced more embarrassment or shame having a deaf child, especially when the child is a girl (36). This difference may partly be explained by the fact that those with a low level of education have less knowledge of child deafness, treatments, and less access to available supportive resources. On the other hand, mothers with a higher level of education are better able to use social facilities and are less prone to stigma (37,38). 

Half of the mothers in this study claimed that other people make fun of them and give them strange looks, and this is consistent with the results of other studies. Mothers are more susceptible than fathers to the response of others to their child’s disabilities and do not tolerate stigmatizing looks (25). Ebadollahi et al. showed that people with disabilities and their families were subjected to various labels, taunts, and sometimes ridicule from society throughout their lives; which causes a sense of futility and worthlessness (12). 

## Conclusions

Overall, the findings of this study show that mothers with deaf children face a high level of stigma in society. A significant number of mothers experienced scorn, pity, and strange looks, and felt ashamed. Thus, education of mothers and the wider society may be effective in increasing awareness and reducing the stigma experienced by mothers with a deaf child. 

## Limitations

This study was conducted among mothers of a deaf child referring to rehabilitation centers in Ardabil Welfare Organization. Due to the relatively small sample, more studies are required to generalize these results to the entire population of mothers. Furthermore, the retrospective nature of the mothers’ responses to questions about the deaf child may be affected by recall bias. 
